# Photon-counting computed tomography: ‘one-stop shop’ for coronary stenosis, inflammation, and myocardial assessment in ST-segment elevation acute coronary syndrome

**DOI:** 10.1093/ehjci/jeae003

**Published:** 2024-01-09

**Authors:** Rafail A Kotronias, Betty Raman, Vanessa Ferreira, Stefan Neubauer, Charalambos Antoniades

**Affiliations:** Acute Multidisciplinary Imaging and Interventional Centre (AMIIC), Division of Cardiovascular Medicine, Radcliffe Department of Medicine, University of Oxford, West Wing L6, John Radcliffe Hospital, Headley Way, Oxford OX3 9DU, UK; Acute Multidisciplinary Imaging and Interventional Centre (AMIIC), Division of Cardiovascular Medicine, Radcliffe Department of Medicine, University of Oxford, West Wing L6, John Radcliffe Hospital, Headley Way, Oxford OX3 9DU, UK; Acute Multidisciplinary Imaging and Interventional Centre (AMIIC), Division of Cardiovascular Medicine, Radcliffe Department of Medicine, University of Oxford, West Wing L6, John Radcliffe Hospital, Headley Way, Oxford OX3 9DU, UK; Acute Multidisciplinary Imaging and Interventional Centre (AMIIC), Division of Cardiovascular Medicine, Radcliffe Department of Medicine, University of Oxford, West Wing L6, John Radcliffe Hospital, Headley Way, Oxford OX3 9DU, UK; Acute Multidisciplinary Imaging and Interventional Centre (AMIIC), Division of Cardiovascular Medicine, Radcliffe Department of Medicine, University of Oxford, West Wing L6, John Radcliffe Hospital, Headley Way, Oxford OX3 9DU, UK

**Figure:**
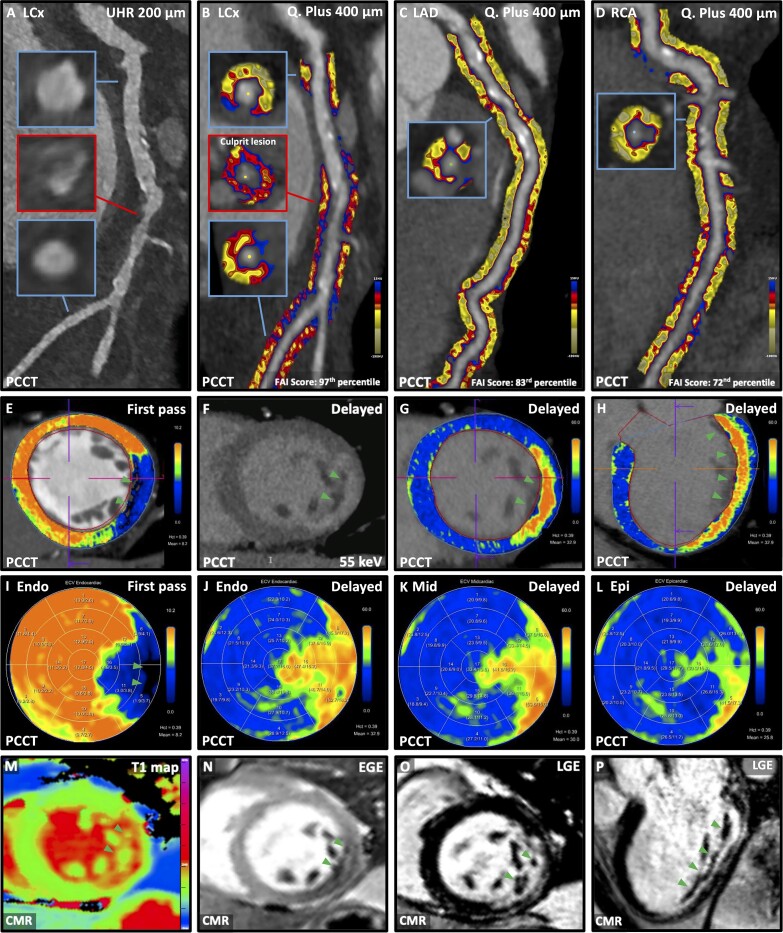


A 69-year-old, asymptomatic, and haemodynamically stable male was admitted 48 h following chest pain with lateral ST-segment elevation acute coronary syndrome (STEACS).

Photon-counting computed tomography angiography (PCCTA) with the NAETOM Alpha (Siemens Healthineers) at ultra-high-resolution revealed a re-canalized non-calcified plaque at the left circumflex (LCx, *Panel A*) and a totally occluded, retrogradely filling right coronary artery (RCA). To confirm the culprit vessel, perivascular fat analysis was performed and fat attenuation index scores were measured (CaRi-Heart® v2.6, Caristo Diagnostics) demonstrating a highly inflamed LCx (*Panel B*) and less inflamed left anterior descending (*Panel C*) and RCA (*Panel D*).

Myocardial extracellular volume (ECV) was assessed on PCCTA at 55 keV (CT Cardiac Functional Analysis 2.1.0 prototype, Siemens Healthineers). First-pass (*Panel E*) and delayed phase (7-min; see Supplementary data online; *Panels F–H*) acquisitions identified a sub-endocardial area of hypodense myocardium and late iodine enhancement in the lateral wall. First-pass iodine-based ECV mapping confirmed microvascular obstruction (MVO, *Panel I*). Transmyocardial polar plots of delayed phase iodine-based ECV mapping showed a lateral wall infarction with 50–75% transmurality (*Panels J–L*).

Cardiac magnetic resonance confirmed the sub-endocardial core of MVO on pre-contrast–native T1 map and early gadolinium enhancement imaging (*Panels M* and *N*) and the 50–75% transmural lateral wall infarction on late gadolinium enhancement imaging (*Panels O* and *P*).

This is the first case of simultaneous assessment of coronary inflammation/plaque and myocardium with PCCTA in a patient with STEACS, revealing the culprit artery and evaluating the myocardial tissue state. Pending clinical validation, PCCTA may provide a practical, one-stop shop for fast comprehensive assessment of acute coronary syndromes, guiding the re-vascularization strategy and long-term management.

## Supplementary Material

jeae003_Supplementary_Data

## Data Availability

The data underlying this article will be shared on reasonable request to the corresponding author.

