# Supplementary material for: Photon-counting computed tomography: ‘one-stop shop’ for coronary stenosis, inflammation, and myocardial assessment in ST-segment elevation acute coronary syndrome
Source: Eur Heart J Cardiovasc Imaging. 2024 Jan 9;25(6):e165. doi: 10.1093/ehjci/jeae003 (PMC11139512; doi:10.1093/ehjci/jeae003)
Supplement: jeae003_Supplementary_Data [file jeae003_supplementary_data.docx]

**Supplement**

The patient was recruited in the **Ox**ford **A**cute **M**yocardial **I**nfarction Study (11/SC0397).

**PCCTA**

In preparation of the PCCTA the patient had 800 mcg of sublingual GTN and 40 mg of intravenous metoprolol. The PCCTA imaging protocol is illustrated in Supplementary Figure 1. Briefly, the PCCT imaging was performed with a first-generation system (NAETOM Alpha, Siemens Healthineers) during index hospitalisation. Quantum Plus acquisitions were acquired with prospective gating (Flash protocol) guided by participant’s heart rate and rhythm, with a gantry rotation time of 0.25s in the axial plane, at a slice thickness of 0.4 mm. This scan was also used for the Fat Attenuation Index (FAI) Score analysis,(1, 2) using a PCCTA-calibrated algorithm, regulatory cleared for clinical use with the label of coronary inflammation index (CaRi-Heart®v2.6, Caristo Diagnostics).

Ultra-high resolution (UHR) acquisitions were retrospectively gated (Flex protocol) with a gantry rotation time of 0.25s in the axial plane at a slice thickness of 0.2 mm. Tube voltage was set at 140kV for the Quantum Plus PCCTA and 120kV for the UHR PCCTA whilst tube current was automatically adjusted to the chosen image quality level of each acquisition (CARE Dose4D, Siemens Healthineers).

A Quantum Plus coronary CT angiogram scan with 70 ml of intravenously administered iodinated contrast media (Omnipaque 350 mgI/ml, GE HealthCare) followed by a saline chaser of 50 ml with a flow rate of 4.5 ml/s was acquired in Flash mode with a care kV image quality of 89 and electrocardiogram triggering after 65% of the RR interval. An ultra-high resolution coronary CTA with 80ml of intravenously administered iodinated contrast media (Omnipaque 350 mgI/ml, GE HealthCare) followed by a saline chaser of 50 ml with a flow rate of 4.5 ml/s was acquired in Flex mode with a care kV image quality of 69 and electrocardiogram triggered window set from 70-80% of the RR interval. Finally, a delayed phase late iodine enhancement Quantum Plus PCCT scan was acquired 7 minutes after the last contrast injection in Flash mode with a care kV image quality of 49 and electrocardiogram triggering after 65% of the RR interval. The total dose length product for the scan was 398 mGy*cm.

**CMR**

Cardiac magnetic Resonance (CMR) imaging was performed using a 1.5T MRI scanner (MAGNETOM Avanto Fit, Siemens Healthcare, Erlangen, Germany) using an 18-channel phased-array coil. Cine images were acquired in three long-axis views (HLA, VLA, LVOT views) and in short-axis slices covering the whole left ventricle (LV) using balanced steady-state free precession cine imaging.(3,4) The Shortened Modified Look-Locker Inversion recovery (ShMOLLI) prototype sequence was used to acquire T1 maps in three short-axis slices reflecting the base, mid and apex of the ventricle.(5,6) In-line quality assessment of ShMOLLI T1-maps was performed using parametric goodness-of-fit (R2) maps at time of acquisition.(6) For late gadolinium imaging, a bolus dose of gadolinium (0.1 mmol/kg intravenous, Gadobutrol, Gadovist, Bayer Schering, Germany) was administered followed by a 15ml saline flush. Early gadolinium images were acquired at 1-2 minutes from the injection of contrast.(7) Late gadolinium enhancement (LGE) imaging (using phase-sensitive inversion recovery imaging) was performed ~8-10 min after the bolus dose in the long axes and short-axis slices covering the left ventricle.(8, 9)

**References**

1. Antoniades C, Tousoulis D, Vavlukis M, Fleming I, Duncker DJ, Eringa E, et al. Perivascular adipose tissue as a source of therapeutic targets and clinical biomarkers. Eur Heart J. 2023;44(38):3827-44.

2. Oikonomou EK, Antonopoulos AS, Schottlander D, Marwan M, Mathers C, Tomlins P, et al. Standardized measurement of coronary inflammation using cardiovascular computed tomography: integration in clinical care as a prognostic medical device. Cardiovasc Res. 2021;117(13):2677-90.

3. Kramer CM, Barkhausen J, Bucciarelli-Ducci C, Flamm SD, Kim RJ, Nagel E. Standardized cardiovascular magnetic resonance imaging (CMR) protocols: 2020 update. Journal of Cardiovascular Magnetic Resonance 2020; 22(1): 17.

4. Schulz-Menger J, Bluemke DA, Bremerich J, et al. Standardized image interpretation and post-processing in cardiovascular magnetic resonance - 2020 update. Journal of Cardiovascular Magnetic Resonance 2020; 22(1): 19.

5. Piechnik SK, Ferreira VM, Dall'Armellina E, et al. Shortened Modified Look-Locker Inversion recovery (ShMOLLI) for clinical myocardial T1-mapping at 1.5 and 3 T within a 9 heartbeat breathhold. J Cardiovasc Magn Reson 2010; 12(1): 69.

6. Ferreira VM, Piechnik SK, Dall'Armellina E, Karamitsos TD, Francis JM, Choudhury RP, et al. Non-contrast T1-mapping detects acute myocardial edema with high diagnostic accuracy: a comparison to T2-weighted cardiovascular magnetic resonance. J Cardiovasc Magn Reson. 2012;14(1):42.

7. Mather AN, Lockie T, Nagel E, et al. Appearance of microvascular obstruction on high resolution first-pass perfusion, early and late gadolinium enhancement CMR in patients with acute myocardial infarction. Journal of Cardiovascular Magnetic Resonance 2009; 11(1): 33.

8. Dall'Armellina E, Ferreira VM, Kharbanda RK, Prendergast B, Piechnik SK, Robson MD, et al. Diagnostic value of pre-contrast T1 mapping in acute and chronic myocardial infarction. JACC Cardiovasc Imaging. 2013;6(6):739-42.

9. Dall'Armellina E, Piechnik SK, Ferreira VM, Si QL, Robson MD, Francis JM, et al. Cardiovascular magnetic resonance by non contrast T1-mapping allows assessment of severity of injury in acute myocardial infarction. J Cardiovasc Magn Reson. 2012;14(1):15.


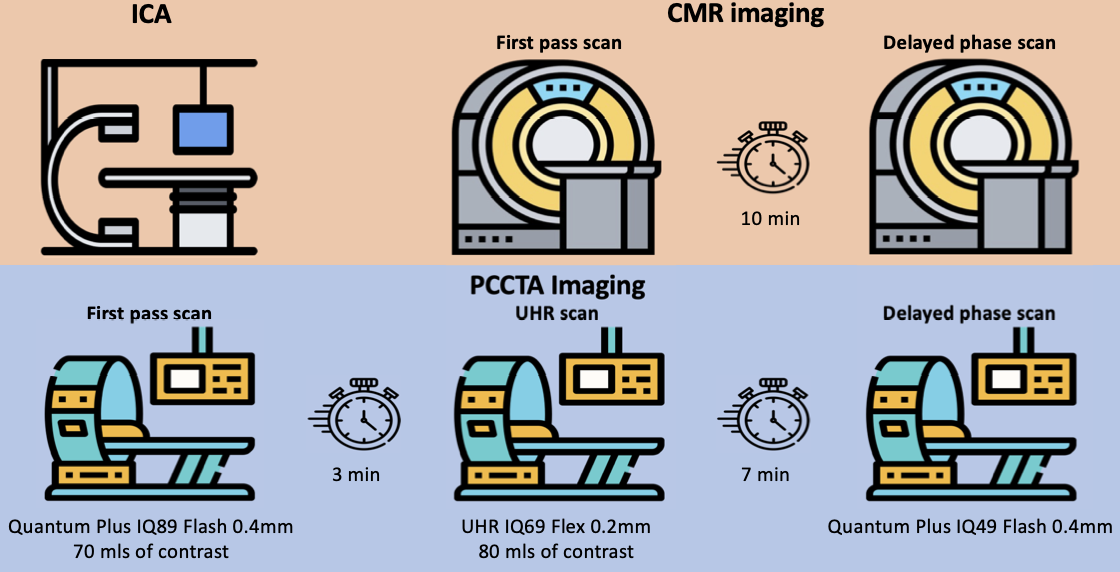
**Supplementary Figure 1**

**Supplementary Figure 1 – Imaging protocol of multimodality coronary and myocardial evaluation with ICA, CMR and PCCTA.** CMR: cardiac magnetic resonance; ICA: invasive coronary angiography; PCCTA: photon counting coronary computed tomography angiography; UHR: ultra-high resolution.

Supplementary Figure 1 acknowledgement: The figure was designed using icons made by [Linector](https://www.flaticon.com/authors/linector), [Freepik](http://Freepik), [eucalyp](https://www.flaticon.com/authors/eucalyp) and Nikita Golubev from [www.flaticon.com](https://www.flaticon.com/)
